# Severe Stercoral Colitis Requiring Extensive Colectomy: A Report of Two Cases

**DOI:** 10.70352/scrj.cr.26-0254

**Published:** 2026-07-07

**Authors:** Mika Naritomi, Katsuhiro Ogawa, Kota Arima, Yukiharu Hiyoshi, Yuji Miyamoto, Yuko Miyasato, Keita Kai, Masaaki Iwatsuki

**Affiliations:** 1 Department of Gastroenterological Surgery, Graduate School of Life Science, Kumamoto University, Kumamoto, Kumamoto, Japan; 2 Department of Diagnostic Pathology, Kumamoto University Hospital, Kumamoto, Kumamoto, Japan

**Keywords:** stercoral colitis, colonic ischemia, indocyanine green fluorescence imaging, total colectomy, surgical decision-making

## Abstract

**INTRODUCTION:**

Stercoral colitis caused by fecal impaction can lead to life-threatening ischemic injury of the colon. However, accurately assessing the severity and reversibility of ischemia remains challenging, particularly when determining the appropriate extent of surgical resection.

**CASE PRESENTATION:**

We report 2 cases of stercoral colitis requiring emergency surgery. In both cases, CT demonstrated marked colonic dilatation due to fecal impaction, and intraoperative findings suggested diffuse colonic ischemia. Indocyanine green (ICG) fluorescence imaging revealed patchy hypoperfusion throughout the colon. Despite similar intraoperative findings, histopathological results differed: ischemic changes were limited to the mucosa in 1 case, whereas transmural necrosis was observed in the other. Both patients presented with severe systemic deterioration and were successfully treated with total colectomy followed by intensive postoperative management.

**CONCLUSIONS:**

In severe stercoral colitis, the extent of histopathological ischemia does not necessarily correlate with clinical severity. Although ICG fluorescence imaging is useful for assessing bowel perfusion, it should not be used as the sole determinant of resection extent. Surgical decision-making should prioritize the overall clinical condition and the extent of colonic involvement, and extensive colectomy may be justified as a life-saving strategy in selected critically ill patients.

## Abbreviation

ICG
: indocyanine green

## INTRODUCTION

Stercoral colitis (also called stercoral proctitis if limited to the rectum) is an inflammatory condition of the bowel wall caused by fecal impaction. The resulting fecaloma leads to focal dilation of the bowel lumen and increased intraluminal pressure. When the pressure compromises vascular perfusion, this may lead to pressure-induced necrosis. If not appropriately managed, this process can progress to transmural necrosis and life-threatening complications.^1–3)^

However, assessing the severity and extent of colonic ischemia remains challenging in clinical practice. Preoperative CT findings, such as bowel dilatation, pneumatosis intestinalis, or pericolic fat stranding, are useful for diagnosis but do not always accurately reflect the severity of ischemic injury.^4)^ Intraoperative macroscopic findings alone may also be insufficient to determine bowel viability, making it difficult to decide the optimal extent of bowel resection.^5)^

Recently, intraoperative ICG fluorescence imaging has been increasingly used to evaluate intestinal perfusion and guide surgical decision-making.^6–8)^ Nevertheless, the relationship between ICG fluorescence findings and the actual severity or depth of ischemic injury has not been fully elucidated, particularly in cases of stercoral colitis associated with fecal impaction.

Herein, we report 2 cases of stercoral colitis caused by fecal impaction, in which preoperative imaging, intraoperative ICG fluorescence assessment, and histopathological findings were directly compared. These cases highlight the limitations of intraoperative assessment of ischemic injury and emphasize the importance of clinical judgment in determining the optimal extent of surgical resection.

## CASE PRESENTATION

### Case 1

**Fig. 1:**
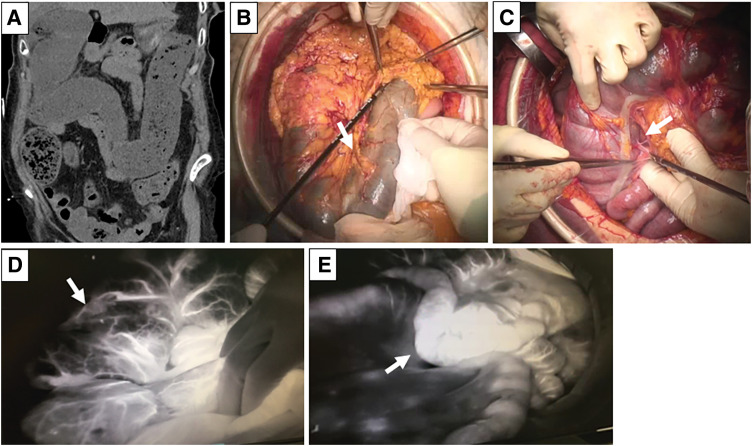
Intraoperative findings and ICG fluorescence imaging in Case 1. (**A**) Abdominal CT showing marked colonic dilatation with fecal impaction. (**B**) Intraoperative macroscopic findings demonstrating necrotic changes with dark discoloration in the transverse colon. (**C**) Macroscopically preserved appearance of the ascending colon. (**D**) ICG fluorescence imaging showing reduced perfusion in the necrotic segments. (**E**) ICG fluorescence imaging demonstrating good perfusion in the small intestine. Arrows indicate macroscopically necrotic segments (**B**), macroscopically preserved colon (**C**), and areas of reduced perfusion on ICG fluorescence imaging (**D**, **E**). ICG, indocyanine green

An 81-year-old woman was referred to our department with acute abdominal pain. Her medical history included ulcerative colitis, for which she had been receiving oral prednisolone (5 mg/day). Five days prior to admission, she had undergone a total mastectomy with axillary lymph node dissection for breast cancer. On presentation, her vital signs were as follows: Glasgow Coma Scale E4V5M6, respiratory rate 29 breaths/min, heart rate 76 beats/min, blood pressure 94/64 mmHg, body temperature 35.5°C, and oxygen saturation 96% on 2 L/min oxygen via nasal cannula. Her quick Sequential Organ Failure Assessment (qSOFA) score was 2. Laboratory findings revealed metabolic acidosis (pH 7.307, HCO_3_^−^ 13.1 mmol/L, base excess −11.6 mmol/L) and an elevated lactate level of 4.17 mmol/L. Markedly elevated liver enzymes (aspartate transaminase 1511 U/L, alanine aminotransferase 453 U/L) and a high procalcitonin level (51.77 ng/mL) were also noted. Abdominal CT showed extensive fecal impaction extending from the ileocecal region to the sigmoid colon, with marked colonic dilatation and pericolic fat stranding. No evidence of neoplastic lesions or mechanical obstruction was observed (**Fig. 1A**). Emergency laparotomy was performed. Intraoperative findings revealed marked dilatation of the entire colon. Macroscopic examination demonstrated necrotic changes with dark discoloration in the transverse colon (**Fig. 1B**), whereas the ascending colon appeared macroscopically preserved (**Fig. 1C**). The small intestine appeared viable. ICG fluorescence imaging demonstrated reduced perfusion in the necrotic colonic segments (**Fig. 1D**). In contrast, good perfusion was observed in the small intestine (**Fig. 1E**). Although **Fig. 1D** shows representative findings in the necrotic areas, patchy hypoperfusion was also observed in other colonic segments that appeared macroscopically viable. Considering the presence of focal necrosis and the possibility of more extensive ischemic involvement suggested by ICG imaging, subtotal colectomy with ileostomy was performed.

Histopathological examination revealed mucosal necrosis and hemorrhagic changes, along with submucosal edema and congestion. However, no necrosis of the muscularis propria was observed, indicating that the ischemic injury had not progressed to irreversible transmural necrosis. Postoperatively, the patient developed septic shock and acute kidney injury requiring continuous hemodiafiltration. She also developed disseminated intravascular coagulation and required transfusion therapy. Her condition gradually improved, and she was weaned from hemodiafiltration on POD 4 and extubated on POD 12. She was transferred to the general ward on POD 20 and subsequently transferred to another hospital for rehabilitation on POD 26.

### Case 2

**Fig. 2:**
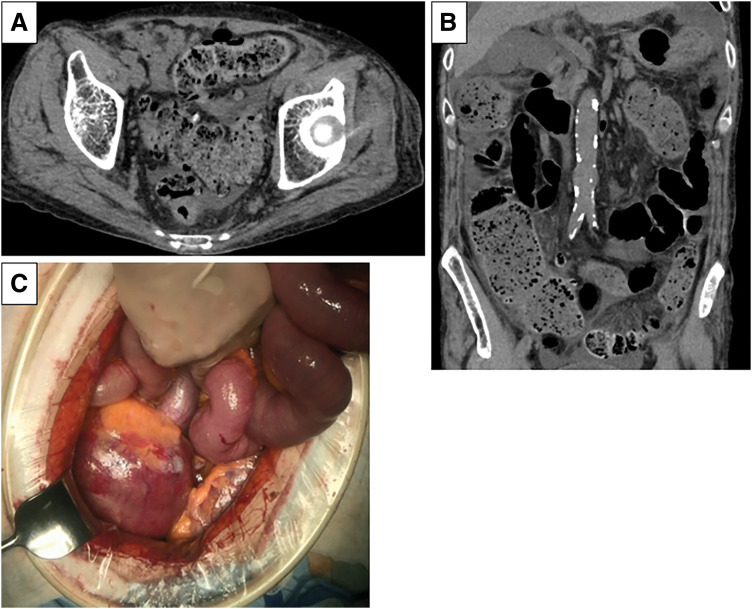
Intraoperative findings in Case 2. (**A**, **B**) CT demonstrated fecal impaction extending from the ileocecal region to the sigmoid colon, with pneumatosis intestinalis in the sigmoid colon. (**C**) Intraoperative findings revealed marked dilatation of the cecum with poor coloration, as well as discoloration around the splenic flexure.

A 76-year-old man was referred to our department with abdominal pain. His medical history included left nephroureterectomy and partial cystectomy for left renal pelvic cancer, chronic obstructive pulmonary disease, atrial fibrillation, and hypertension. He was also receiving high-dose steroid therapy (prednisolone 45 mg/day) for rapidly progressive glomerulonephritis due to anti-glomerular basement membrane disease. He had experienced severe constipation, with minimal bowel movements over several days. Abdominal pain developed and persisted, prompting further evaluation. On admission, his vital signs were as follows: Glasgow Coma Scale E4V5M6, respiratory rate 27 breaths/min, heart rate 123 beats/min, blood pressure 88/56 mmHg, body temperature 36.9°C, and oxygen saturation 96% on room air. His qSOFA score was 2. Laboratory tests revealed a marked inflammatory response (white blood cell count 40000/μL, C-reactive protein 26.39 mg/dL, procalcitonin 8.62 ng/mL) and severe renal dysfunction (blood urea nitrogen 99.0 mg/dL, creatinine 7.54 mg/dL). Arterial blood gas analysis showed pH 7.473 and a lactate level of 1.64 mmol/L. Abdominal CT demonstrated extensive fecal impaction from the ileocecal region to the sigmoid colon, with marked colonic dilatation. Pneumatosis intestinalis was observed in the sigmoid colon (**Fig. 2A** and **2B**). Based on these findings, severe stercoral colitis with suspected bowel ischemia and peritonitis was diagnosed, and emergency surgery was performed. Intraoperatively, marked dilatation and poor coloration of the cecum were observed, along with ischemic changes around the splenic flexure (**Fig. 2C**). ICG fluorescence imaging was also performed, demonstrating reduced perfusion in the right colon including the cecum, as well as around the splenic flexure, consistent with the macroscopic findings. However, fluorescence images were not recorded and were therefore unavailable for retrospective analysis. This reflects a limitation in retrospective data acquisition rather than selective omission. Extensive colonic ischemia was suspected, and total colectomy with abdominal lavage and ileostomy was performed. Histopathological examination revealed transmural necrosis involving the muscularis propria, confirming irreversible ischemic injury. The patient required intensive postoperative care, but his condition gradually improved. He was transferred back to the referring hospital on POD 48.

## DISCUSSION

Stercoral colitis is a relatively rare but potentially life-threatening condition caused by increased intraluminal pressure due to fecal impaction, leading to impaired colonic blood flow. As ischemia progresses, it may result in transmural necrosis, perforation, and sepsis, all of which are associated with high mortality.^1–3,9)^ Notably, previous reports have suggested that non-perforated stercoral colitis complicated by septic shock may be associated with even higher mortality than perforated cases, highlighting the importance of early recognition and prompt intervention.^10)^

**Fig. 3:**
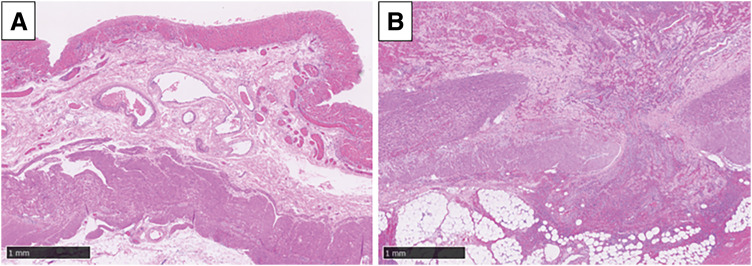
Histopathological findings. (**A**) Case 1: Ischemic changes limited to the mucosal layer, with preservation of the muscularis propria. (**B**) Case 2: Transmural necrosis involving the muscularis propria, consistent with irreversible ischemic injury. Hematoxylin and eosin staining (original magnification ×20).

A major challenge in the management of stercoral colitis is determining the appropriate extent of bowel resection. Although histopathological evaluation can distinguish between reversible and irreversible ischemia, this information is not available intraoperatively. Therefore, surgeons must rely on a combination of preoperative imaging, intraoperative findings, and the patient’s systemic condition. However, ischemic changes in stercoral colitis are often patchy and heterogeneous, making it difficult to accurately identify viable bowel segments. In the present cases, both patients demonstrated extensive fecal impaction and diffuse colonic dilatation, accompanied by severe systemic deterioration. Intraoperative findings suggested widespread ischemia, and ICG fluorescence imaging revealed patchy hypoperfusion throughout the colon. Despite these similar findings, histopathological results differed markedly between the 2 cases: ischemic changes were limited to the mucosa in Case 1, whereas transmural necrosis was observed in Case 2 (**Fig. 3**). These findings suggest that the degree of histopathological ischemia does not always correlate with the clinical severity or intraoperative assessment. Even in the absence of transmural necrosis, patients may present with severe systemic manifestations such as septic shock, indicating clinically significant ischemia. In such situations, limited resection based solely on presumed reversibility may be insufficient. Residual ischemic segments may persist and lead to ongoing sepsis, delayed necrosis, or subsequent perforation.^3)^ In Case 1, histopathological examination revealed ischemia limited to the mucosal layer, raising the possibility that the extent of resection may have been greater than necessary. Although extensive colectomy was performed to avoid leaving potentially ischemic bowel in the setting of diffuse involvement and severe clinical deterioration, the risk of overtreatment should be carefully considered. Given the morbidity associated with extensive colectomy, determining the optimal extent of resection in such cases remains a significant clinical challenge. This highlights the inherent difficulty in balancing the risk of overtreatment against the risk of leaving residual ischemic bowel. The 2 cases presented in this report differed substantially in clinical background, including immunosuppression status and overall systemic condition. These factors may have influenced both the progression of ischemia and postoperative outcomes. Therefore, direct comparison between the 2 cases is limited, and conclusions drawn from this comparison should be interpreted with caution.

To better contextualize our findings, we performed a literature review using PubMed. Articles were searched using the keyword “stercoral colitis,” and previously reported cases requiring surgical intervention were identified and reviewed. Cases with available information on surgical procedures, presence of perforation, and clinical outcomes were included in the analysis (**Tables 1**^1,4,9–25)^ and **2**). As this review was limited to cases requiring surgical intervention, the included patients predominantly represented advanced disease, often with diffuse colonic involvement, which may explain the need for extensive resection in selected cases (**Table 1**). In this review, all patients who underwent subtotal or total colectomy survived (6/6, 100%), whereas mortality was observed among patients who underwent limited resection (3/12, 25.0%) (**Table 2**). These findings may be influenced by selection bias, as patients undergoing extensive colectomy may have been more aggressively managed or selected based on intraoperative judgment. Although these findings suggest a potential association between the extent of resection and patient outcomes, they should be interpreted with caution due to the small sample size and the retrospective nature of the analysis. Information regarding validated severity scores, such as Acute Physiology and Chronic Health Evaluation II (APACHE II) or SOFA, was not consistently reported in the reviewed cases. Therefore, an objective comparison of disease severity between patients undergoing extensive colectomy and those undergoing limited resection was not feasible. This lack of standardized severity assessment represents an important limitation of the current literature and may contribute to selection bias, as patients undergoing surgery likely represent a subset of more clinically severe cases. Therefore, our findings should be interpreted with caution and viewed as generating hypotheses rather than establishing definitive surgical strategies. Previous studies have also highlighted the risk associated with limited resection in stercoral colitis. Segmental colectomy may fail to remove all affected bowel, particularly in cases with diffuse involvement, and has been associated with poor outcomes due to residual disease. In contrast, more extensive procedures, such as subtotal or total colectomy, may reduce the risk of residual ischemic colon and improve outcomes in selected patients with severe disease.^4,11)^

**Table 1:** Summary of reported surgical cases of stercoral colitis

Age (years)	Sex	Surgery performed	Subtotal/total colectomy	Perforation	Outcome	Ref.
79	Female	Yes	No	Yes	Unknown	^1)^
80	Male	Yes	No	No	Survival	^4)^
55	Female	Yes	No	Yes	Survival	^9)^
36	Male	Yes	No	No	Survival	^10)^
78	Female	Yes	No	Yes	Death	^11)^
56	Female	Yes	No	Unknown	Death	^13)^
80	Female	Yes	Yes	No	Survival	^14)^
78	Female	Yes	No	Unknown	Death	^15)^
57	Male	Yes	No	Yes	Survival	^16)^
46	Male	Yes	Yes	No	Survival	^17)^
28	Female	Yes	No	No	Survival	^18)^
35	Male	Yes	Yes	Yes	Survival	^19)^
80	Female	Yes	Yes	No	Survival	^20)^
60	Female	Yes	Yes	No	Survival	^21)^
65	Female	Yes	No	Yes	Survival	^22)^
13	Male	Yes	No	Yes	Survival	^23)^
45	Female	Yes	No	No	Unknown	^24)^
72	Male	Yes	Yes	No	Survival	^25)^

Cases are presented in the order of reference as cited in the text.

Ref., reference number

**Table 2:** Summary of reported surgical cases of stercoral colitis and outcomes according to extent of resection

Variable	Subtotal/total colectomy (n = 6)	Limited resection (n = 12)
Survival	6 (100%)	7 (58.3%)
Death	0 (0%)	3 (25.0%)
Unknown	0 (0%)	2 (16.7%)

Subtotal/total colectomy was defined as resection of the majority or entirety of the colon. Limited resection included segmental colectomy or partial resection. Outcomes were categorized as survival, death, or unknown based on available reports. Percentages were calculated based on available data.

ICG is a water-soluble fluorescent dye that binds to plasma proteins and emits near-infrared fluorescence when excited by a specific wavelength of light, allowing real-time visualization of tissue perfusion. In the present cases, ICG (Diagnogreen, 25 mg; Daiichi Sankyo, Tokyo, Japan) was dissolved in 10 mL of normal saline, and 3 mL was administered intravenously as a bolus. Fluorescence imaging was performed using a near-infrared camera system (Photodynamic Eye; Hamamatsu Photonics, Shizuoka, Japan). The time to fluorescence appearance was not formally measured; however, fluorescence was observed promptly after intravenous administration in both cases, and assessment was based on qualitative visual evaluation. In both cases, ICG fluorescence imaging demonstrated areas of reduced or absent perfusion; however, its contribution to surgical decision-making differed between the 2 cases. Importantly, no clear difference in fluorescence patterns was observed that could distinguish between mucosal ischemia and transmural necrosis, indicating that ICG does not provide information regarding the depth or reversibility of ischemic injury. In addition, quantitative assessment of fluorescence intensity was not performed, and dosing was not adjusted according to body weight, representing further methodological limitations.

In Case 1, ICG fluorescence imaging revealed additional areas of perfusion abnormality that were not clearly appreciated on macroscopic inspection alone. These findings contributed to the recognition of more extensive colonic involvement and supported the decision to perform a subtotal colectomy. In contrast, in Case 2, surgical decision-making was primarily driven by clinical severity and macroscopic findings, and the contribution of ICG was more limited. These observations suggest that the clinical utility of ICG fluorescence imaging may vary depending on the stage and extent of disease.

Importantly, the depth of ischemic injury cannot be reliably determined intraoperatively, which complicates surgical decision-making. In our cases, both colons showed dark discoloration macroscopically; however, histopathological findings differed, with mucosal necrosis in 1 case and transmural necrosis in the other. This suggests that macroscopic findings alone do not reliably reflect the depth of ischemic injury. Intraoperative ICG fluorescence imaging can demonstrate perfusion abnormalities but does not allow assessment of tissue viability or reversibility.^25)^ In addition, endoscopic evaluation is limited to the mucosal layer and cannot provide information on deeper structures such as the muscularis propria. In this context, intraoperative ICG fluorescence imaging should be understood as a tool for assessing the extent and distribution of perfusion abnormalities rather than the depth of ischemic injury. It enables rapid and global evaluation of colonic perfusion and may facilitate the detection of ischemic changes that would otherwise only be identified by endoscopic assessment of the mucosa. This makes ICG a practical adjunct in the intraoperative setting. However, it cannot differentiate between reversible mucosal ischemia and irreversible transmural necrosis, and therefore should not be used as the sole determinant of resection extent. Therefore, ICG fluorescence imaging may be useful for delineating the extent of ischemia, but not for determining its depth or reversibility. Given these limitations, surgical decision-making should prioritize the overall clinical condition and the extent of colonic involvement rather than attempting to predict histopathological reversibility. Particularly in cases of diffuse colonic ischemia, as seen in stercoral colitis, even mucosal ischemia may represent clinically significant and potentially progressive injury. In critically ill patients with widespread disease, a more aggressive surgical approach, including subtotal or total colectomy, may be justified as a life-saving strategy. Stercoral colitis does not uniformly require surgical intervention, nor does it mandate extensive colectomy in all cases. Surgical indication should be determined based on the patient’s clinical condition, including hemodynamic instability, elevated lactate levels, and signs of peritonitis. Intraoperatively, the extent of bowel involvement is a key determinant of surgical strategy. Subtotal or total colectomy may be considered in cases with diffuse colonic dilatation and transmural necrosis, or when segmental transmural necrosis is accompanied by extensive ischemic involvement, as suggested by intraoperative findings, including ICG fluorescence imaging. In such situations, limited resection may risk leaving behind compromised bowel, potentially leading to ongoing sepsis or delayed perforation. In contrast, intraoperative endoscopic evaluation is often impractical in stercoral colitis. The presence of extensive fecal impaction and marked colonic distension makes bowel preparation impossible and limits endoscopic visualization, rendering this approach technically challenging and of limited utility in the acute surgical setting. These considerations highlight the importance of individualized surgical decision-making in stercoral colitis.

This study has several limitations. First, the number of cases is small, and ICG fluorescence imaging findings could not be quantitatively evaluated. Second, fluorescence images were not available for both cases, precluding direct comparison. Nevertheless, the direct comparison between intraoperative findings, ICG assessment, and histopathological results provides valuable insights into the complexity of surgical decision-making in stercoral colitis.

In summary, our cases highlight that surgical decision-making in severe stercoral colitis should not rely solely on perfusion imaging or presumed histopathological reversibility. Instead, a comprehensive assessment incorporating clinical severity and the extent of disease is essential. In critically ill patients with diffuse involvement, extensive colectomy may be considered as a life-saving strategy in selected patients.

## CONCLUSIONS

Although ICG fluorescence imaging is useful for assessing colonic perfusion, it does not allow reliable evaluation of the depth or reversibility of ischemic injury. Surgical decision-making should therefore not rely solely on intraoperative perfusion findings. Given the limited number of cases, our findings should be considered exploratory and hypothesis-generating. While extensive resection may represent a reasonable option in selected patients with diffuse disease, careful individualized decision-making remains essential.

## DECLARATIONS

### Availability of data and materials

The datasets supporting the conclusions of this article are included within the article. Additional data are available from the corresponding author on reasonable request.
